# Angularly Cascaded Long-Period Fiber Grating for Curvature and Temperature Detection

**DOI:** 10.3390/s24010184

**Published:** 2023-12-28

**Authors:** Anping Xiao, Jie Du, Qiang Ling, Yao Chen, Zhengtian Gu, Haiyun Chen, Zhangwei Yu, Barerem-Melgueba Mao, Zuguang Guan, Daru Chen

**Affiliations:** 1 Hangzhou Institute of Advanced Studies, Zhejiang Normal University, 1108 Geng Wen Road, Hangzhou 311231, China; 2 Laboratory of Photo-Electric Functional Films, College of Science, University of Shanghai for Science and Technology, 516 Jun Gong Road, Shanghai 200093, China; 3 Futong Group Co., Ltd., Hangzhou 311499, China; 4 College of Science, Zhejiang University of Technology, Hangzhou 310023, China; 5 Key Laboratory of Optical Information Detection and Display Technology of Zhejiang, Zhejiang Normal University, Jinhua 321004, China; 6 Centre Informatique et de Calcul, Universite de Lome, Lome BP 1515, Togo

**Keywords:** optical fiber sensor, long period fiber grating, curvature

## Abstract

A high-sensitivity curvature sensor with dual-parameter measurement ability based on angularly cascaded long-period fiber grating (AC-LPFG) is proposed and experimentally demonstrated, which consists of two titled LPFGs (TLPFGs) with different tilt angles and the same grating period. AC-LPFG was fabricated by using a deep ultraviolet laser and an amplitude-mask in our laboratory. The experimental results show that simultaneous measurement of curvature and temperature can be achieved by monitoring the wavelengths of two resonant peaks for different TLPFGs. The two peaks show opposite shifts with increasing curvature and has a maximum curvature sensitivity of 16.392 nm/m^−1^. With the advantages of low cost, high sensitivity, and dual-parameter measurements, our sensor has more potential for engineering applications.

## 1. Introduction

Long-period fiber gratings (LPFGs) couple the fundamental core mode with several forward-propagating cladding modes, forming a series of loss dips at resonant wavelengths in the transmission spectrum. Due to the high response of the cladding mode to change in the environment, LPFGs have higher sensitivity than other types of optical fiber grating [1]. Recently, R&D LPFG-based sensors have been developed to measure many environmental parameters closely related to our daily lives, such as strain, force, torsion, curvature, magnetic field, surrounding refractive index, and so on [2,3,4]. Regardless of the type of parameter being measured, the temperature crosstalk limits the practical application of LPFG-based sensors.

Especially in curvature measurement, the temperature crosstalk is the spotlight in the R&D of the optical fiber sensors [5]. Some researchers have focused on the temperature-insensitive sensors [6,7] and the compensation method based on a cascaded structure with a low-curvature sensitivity temperature sensor [8] for the curvature sensor design. Further, there are many studies on the simultaneous measurement of curvature and temperature. Examples of these sensors are fiber-optic interferometers and fiber gratings. For interferometer-type curvature sensors, the curvature sensitivity is relatively low, especially for Fabry–Perot interferometers (FPI) [9] and multimode interferences [10]. Weihao Yuan [11] and Fang Zhang et al. [12] proposed a simple Mach–Zehnder interferometer (MZI) using ring core fiber (RCF) for curvature and temperature sensing. The MZI is fabricated by splicing a segment of RC-FMF between two pieces of single-mode fiber (SMF) or no-core fiber (NCF). The max curvature sensitivity of the simple sensor can reach −3.68 nm/m^−1^ in the range from 1.3856 to 3.6661 m^−1^. By utilizing the 2 × 2 matrix algorithm, the dual demodulation of temperature and curvature can be readily realized. Furthermore, the hollow square core fiber (HSCF)-based optical fiber interferometer [13] has been used to measure curvature, temperature, and strain simultaneously. For such a sensor, the antiresonance (AR), MZI, and the cladding modal interferometer (CMI) have been excited by the SMF-HSCF-SMF structure. And various dips for different interferometers could be used to measure dual parameters by utilizing the 3 × 3 matrix algorithm. A maximum curvature sensitivity of 0.63 nm/m^−1^ can be achieved using the CMI. To improve the curvature sensitivity, some fiber-optic processing methods for interference enhancement have been proposed one after another. Min Zhao et al. [14] demonstrated an MZI based on the sandwich structure of the deformed multi-mode fiber (D-MMF) in SMFs. The MMF is deformed by CO_2_ laser etching and hydrogen–oxygen flame melting method. And the deformed MZI structure presents the highest curvature sensitivity reaching up to −23.2 nm/m^−1^ and has the ability to measure dual parameters. Although this type of structural failure can effectively improve sensitivity, its fragile mechanical structure limits its application scenarios.

Similar to the MZI-type sensor, the response sensitivity of LPFG is closely related to the writing technology. Here, we divide the writing methods into destructive-type methods and non-destructive-type methods based on whether the fiber structure is deformed. For the destructive-type methods, hydrogen–oxygen flame heating methods [15] and CO_2_ laser polishing method [16] have been reported to fabricate the curvature sensor. Chupeng Lu et al. [17] reported an arc-remodified LPFG (AR-LPFG) to monitor the changes in the curvature using the CO_2_ laser polishing method; the AR-LPFG contains a conspicuous performance for bending-variation sensing with a maximum sensitivity of 17.02 nm/m^−1^. Xiaofei Li et al. [18] have proposed C-shaped core long-period fiber gratings (C-LPFGs) with two resonant peaks for curvature temperature and temperature sensing fabricated using the methods CO_2_ laser polishing and oxyhydrogen-flame heating. The two peaks show curvature and temperature sensitivities of −24.01/−21.3 nm/m^−1^ and 64/33 pm/°C, respectively. For non-destructive-type methods, the UV exposure method and femtosecond-laser direct-writing method are two traditional LPFG writing methods. The UV exposure method has been used to write multi-core fiber (MCF)-based LPFGs for curvature sensing by David Barrera et al. [19]. The MCF-LPFG sensor shows a linear response for curvature magnitudes from 0 to 1.77 m^−1^ with a maximum sensitivity of −4.85 nm/m^−1^. Additionally, Xinran Dong et al. [20] have fabricated LPFGs using the femtosecond-laser direct-writing method for curvature measurement with a maximum curvature sensitivity of −4.82 nm/m^−1^. Obviously, the LPFG sensors fabricated using the non-destructive-type methods show lower curvature sensitivities than that of destructive-type methods, but they have good mechanical properties to make them more competitive in harsh environmental application. To improve the curvature sensitivity of the LPFG fabricated by non-destructive-type methods, a novel curvature sensor has been developed in our paper.

In this paper, a novel optical fiber sensor based on the angularly cascaded LPFG (AC-LPFG) structure for simultaneous measurement of curvature and temperature has been reported. The sensor is fabricated by illuminating the fiber core with the aid of a deep ultraviolet laser through an amplitude-mask. Through the curvature and temperature tests of our sensor, two resonant peaks show different curvature and temperature responses. By utilizing a 2 × 2 matrix of the sensitivities, the dual-parameter simultaneous measurements can be achieved.

## 2. Fabrication and Principle

**Figure 1:**
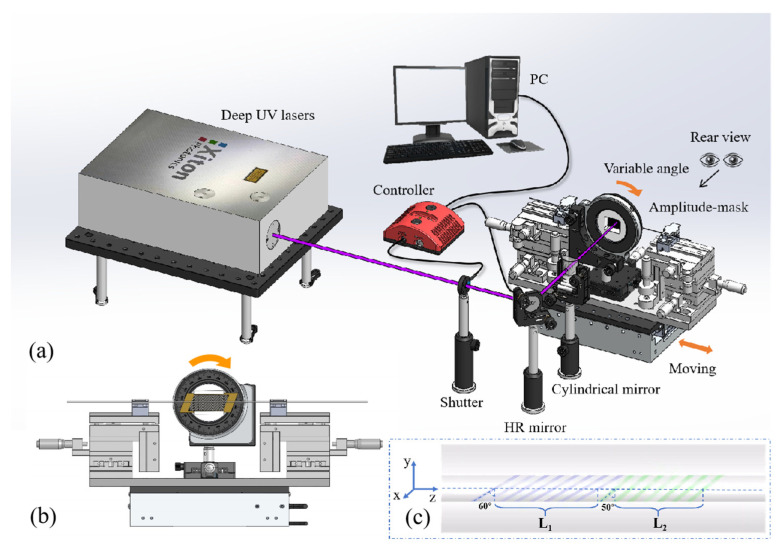
(**a**) The diagram of the AC-LPFG fabricating device, (**b**) enlarged view of the back of the fiber placement device, and (**c**) the diagram of the AC-LPFG structure.

The fiber used to write TLPFG in the experiment is the single-mode fiber (SMF-28e^+^) belonging to Corning company. The parameters of the SMF are as follows: the core diameter is 8.3 μm, the cladding diameter is 125 μm, and the refractive index of the core is 1.4681 at a wavelength of 1550 nm. To increase the photosensitivity, the SMF needs to carry hydrogen for about two weeks in a closed device with high temperature, high pressure, and high concentration of hydrogen. The schematic diagram of the AC-LPFG fabricating setup is shown in Figure 1. In the experiment, the two ends of the hydrogen-loaded SMF are connected with an optical spectrum analyzer (OSA, Yokogawa (Musashino City, Japan), AQ6375, 1200–2400 nm) and a broadband light source (BBS, Fiberer (Shenzhen, China)*,* SLED Light Source, 1250–1650 nm) respectively. In the process of grating writing, the fiber should be clamped by two jigs on a five-dimensional displacement table, so that the fiber is in a stretched state. The deep ultraviolet laser is provided by Xiton Photonics company (Kaiserslautern, Germany) with the maximum average output power of 150 mW and wavelength of 213 nm. The HR (high reflective) mirror is provided by Laseroptik company (Garbsen, Germany). The deep ultraviolet light emitted from the laser first passes through the shutter, then reflects through the mirror, and finally focuses on the fiber core through the cylindrical mirror, wherein the focal length of the cylindrical mirror is 150 mm (Figure 1a). The fiber is placed behind the mask, and the distance between the mask and the fiber is changed by a one-dimensional displacement table installed in the middle of the transfer plate to ensure that the fiber and the mask are close enough but not in contact. The inclined amplitude-mask is placed in the rotary fixture, and the inclination angle of the mask can be changed by the rotary fixture (Figure 1b). The amplitude-mask with an axial period of 494 μm is made of metal, and the rest parts are opaque except the gap.

In the process of writing AC-LPFG, the precise stepping displacement platform (Newport, (Irvine, CA, USA) XMS100-S, Minimum Incremental Motion: 1 nm) controls the fiber to move in the vertical direction of the beam, while the shutter (Thorlabs (Newton, NJ, USA), SHB025T, Ø1/4 Diaphragm Shutters with Controller) has been designed to facilitate rapid switching on and off of the laser light path located at the back end. Both the precise stepping displacement platform and the controller are controlled by PC. When the beam sweeps the fiber, the periodic refractive index changes on the fiber due to the existence of the mask, and the grating length is ensured by the displacement platform. During the process of inscribing the fiber grating, the laser beam is kept motionless. While the fiber and mask plate are fixed on the displacement platform, the software on the PC side controls their position and distance. In this process, the optical fiber and mask plate remain relatively static. The fabrication process of AC-LPFG can be divided into two steps: firstly, the tilt angle is adjusted to 60° and the grating length L_1_ is 1.5 cm; then, the tilt angle is adjusted to 50°, and the second grating is made directly next to the first grating, whose grating length L_2_ is also 1.5 cm. Throughout the process, the length of each LPFG and the spacing between two LPFGs are fully controllable due to the minimum displacement increment of the displacement stage at 1 nm with an accuracy of ±0.75 µm. After the movement is completed, AC-LPFG is formed. The structural diagram of AC-LPFG is given (Figure 1c).

The transmission characteristics of TLPFG can be explained by coupled-mode theory. The coupled-mode equations of TLPFG are [21]:

(1) $$\frac{dA_{LP_{01}}}{dz}=if_{LP_{01}\mathrm{-L}P_{01}}A_{LP_{01}}+i\sum_{l,\nu }g_{cl,l\nu \mathrm{-L}P_{01}}^{+}A_{cl,l\nu }\times \exp\left(-2i\delta_{cl,l\nu \mathrm{-L}P_{01}}z\right),$$

(2) $$\sum_{l,\nu }\left[\frac{dA_{cl,l\nu }}{dz}=+ig_{cl,l\nu \mathrm{-L}P_{01}}^{-}A_{LP_{01}}\times \exp\left(2i\delta_{LP_{01}\mathrm{-c}l,l\nu }z\right)\right],$$

where, $A_{LP_{01}}$ and $A_{cl,l\nu }$ are the amplitudes of the fiber core mode and the l-order νth cladding mode in the +z direction, respectively, $f_{LP_{01}\mathrm{-L}P_{01}}$ is the self-coupling coefficients between the forward fiber core modes, $g_{cl,l\nu \mathrm{-L}P_{01}}^{\pm }$ is the mutual coupling coefficient between the core modes and the l-order νth cladding modes, and $\delta_{LP_{01}\mathrm{-c}l,l\nu }$ is the phase demodulation parameter between the forward core modes and the forward l-order νth cladding modes. According to Ref. [21], $\delta_{LP_{01}\mathrm{-c}l,l\nu }=0$ is the phase-matching condition, and the phase matching equation is as follows:

(3) $$\delta_{LP_{01}\mathrm{-c}l,l\nu }=\frac{1}{2}\left(\beta^{LP_{01}}-\beta_{l\nu }^{cl}-\frac{2\pi }{\Lambda_{L}}\right),$$

where $\beta^{LP_{01}}$ and $\beta_{l\nu }^{cl}$ are the propagation constants of the core mode $LP_{01}$ and the lν cladding mode, respectively. For a TLPFG, the axial grating period can be expressed as:

(4) $$\Lambda_{L}=\frac{\Lambda_{g}}{\cos\theta },$$

where θ is the angle between the grating and the z axis, whose relation with the angle of inclination *α* appearing in the text is as follows: $\theta =\frac{\pi }{2}-\alpha$. And $\Lambda_{g}$ and $\Lambda_{L}$ are the normal grating period and the axial grating period in TLPFG, respectively. 

**Figure 2:**
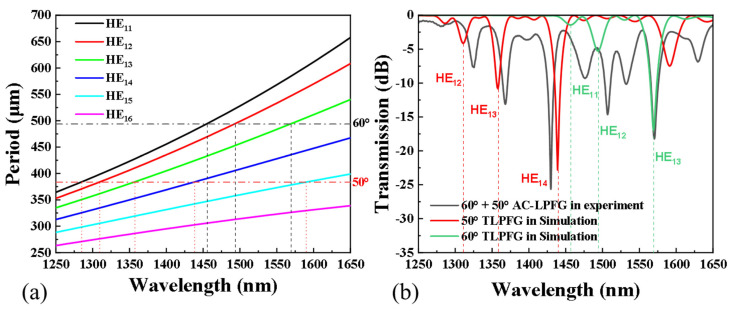
(**a**) PMCs of core mode and cladding mode in different axial periods and (**b**) transmission spectra of TLPFG at 50° and 60° angles and the AC-LPFG.

**Figure 3:**
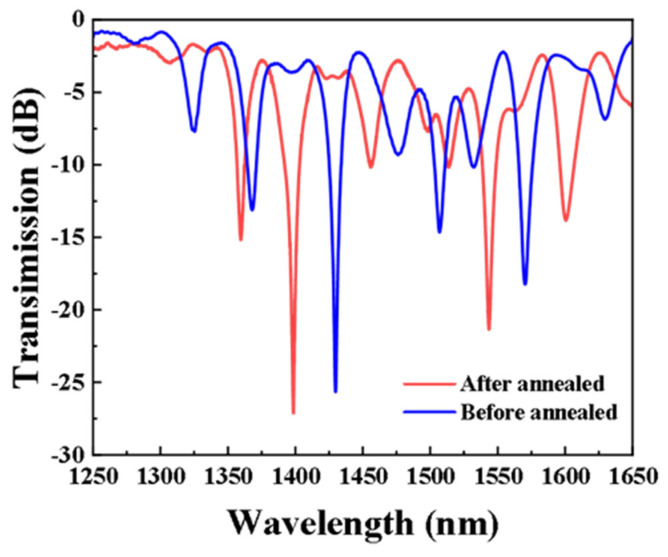
Comparison of transmission spectra before and after annealing.

According to the phase-matching conditions, we simulated phase-matching curves (PMCs) of the core mode and cladding mode (lν) in different axial periods (Figure 2a). And several resonant peaks were obtained corresponding to the tilt angles of 50° and 60°, respectively. The transmission spectra of TLPFG at 50° and 60° angles are simulated according to the coupled-mode equation and compared with the AC-LPFG in the experiment (Figure 2b). It is concluded that the simulation results are basically consistent with the experimental results. By comparison, it can be seen that the resonance peak near 1425 nm is caused by the coupling of the cladding mode HE_14_ and core mode of TLPFG with an inclined angle of 50°, while the resonance peak near 1470 nm is caused by the coupling of the cladding mode HE_13_ and core mode of TLPFG with an inclined angle of 60°. To make the sensor’s sensing performance stable, the sensor made in the laboratory needs to be annealed at last. The sensor was annealed at 100 °C for 8 h, and the comparison of transmission spectra of the sensor before and after annealing is shown (Figure 3). The transmission spectrum has changed greatly before and after annealing. The annealing operation accelerates the escape of unreacted hydrogen molecules from the fiber, which leads to a reduction in the effective refractive index of the fiber. Accordingly, the central wavelength of the resonance peak within the transmission spectrum is altered.

## 3. Experimental Results

### 3.1. Experimental Setup

**Figure 4:**
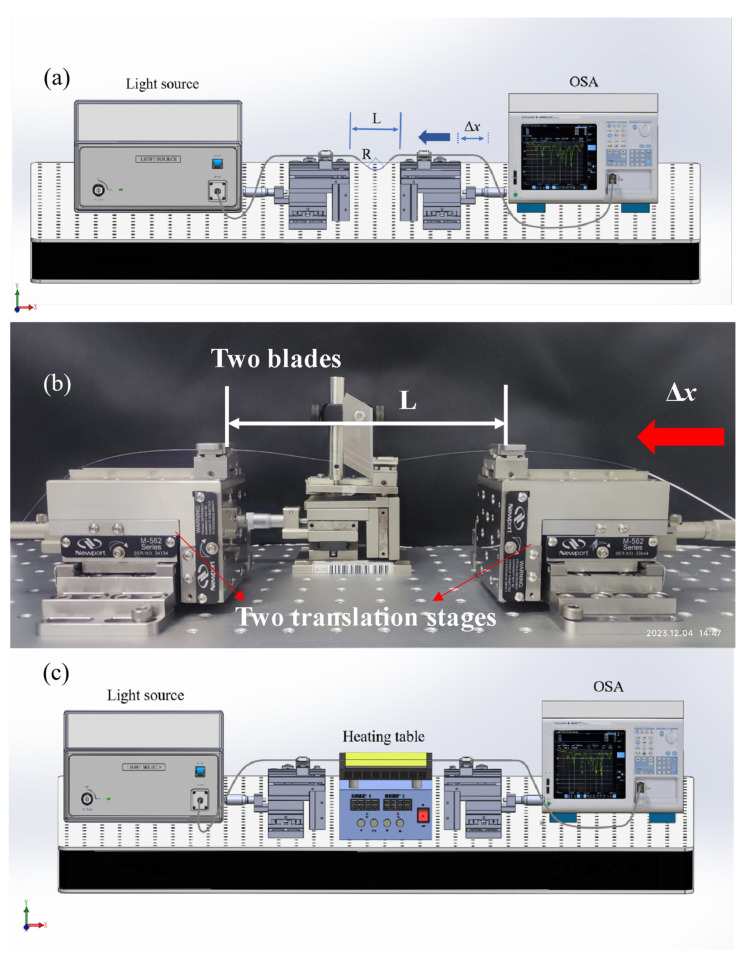
(**a**)The schematic diagram and (**b**) the photograph of the bending experimental setup, and (**c**) the schematic diagram of the temperature-sensing experiment device for AC-TLPFG sensor.

To study the bending response of the sensor, a series of experiments were studied with the setup that is shown (Figure 4a,b). The fiber is fixed on the clamps of two five-dimensional displacement tables (be careful not to stretch the optical fiber too much or relax it, so that it is in a natural stretching state), and at the same time, it is ensured that the clamps on both sides are consistent in the Y direction and Z direction. Additionally, to ensure that the optical fiber is bent only in a single direction in the setup, a narrow gap ~150 μm wide was created with two blades, so as to keep the optical fiber bent in the narrow gap. By moving the displacement table to one end, the fiber can be bent into different curvatures. The applied curvature can be expressed as:

(5) $$C=\frac{1}{R}\approx \sqrt{\frac{24\times \Delta x}{L^{3}}},$$

where R is the radius of curvature at the bend, L is the initial distance between the two ends, and Δx is the distance that the fixture at one end moves inward.

In the temperature-sensing test part, the sensor is placed on a heating table (Figure 4b), and its temperature range is set at 25 °C to 100 °C, with a step of 10 °C. It is necessary to position the optical fiber evenly close to the heating table to ensure the accuracy of the experimental results.

### 3.2. Curvature Sensitivity

During the experiment, the initial length L of both ends of the fixture is 151 mm (Figure 4a). To ensure that the initial curvature is zero, during placement of the sensor, a weak tensile force is applied to the sensor to ensure that it is in a straight state. The displacement table on the left side remains motionless, and the displacement table on the right side moves inward in the X direction, with the movement ranging from 0 to 0.12 mm and the corresponding curvature ranging from 0 to 0.915 m^−1^.

**Figure 5:**
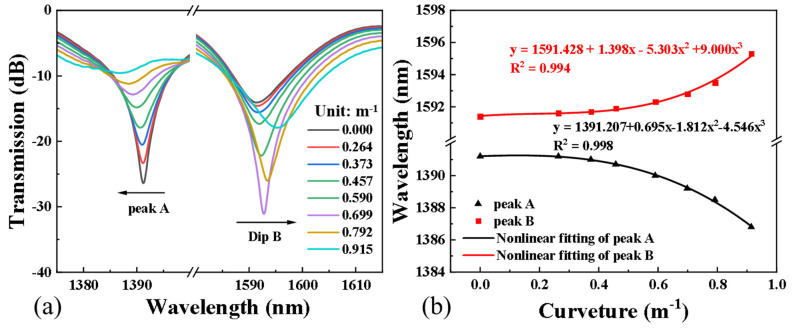
(**a**) The transmission spectrum of the sensor and (**b**) the changes in the wavelength and the fitting results of two resonance peaks in the curvature range of 0 to 0.915 m^−1^.

The transmission spectral response with increasing curvatures has been measured at room temperature with the wavelength ranging from 1250 nm to 1650 nm (Figure 5a). There are many resonant peaks in the transmission spectrum; for the comparison, only two representative resonant peaks were intercepted for analysis. With the increase in curvature, peak A has an obvious blue shift, while peak B has a red shift, and the transmission spectrum depth increases first and then decreases (Figure 5a). Figure 5b shows the corresponding change in the wavelength of peak A and peak B in the curvature range of 0 to 0.915 m^−1^, with the measured data fitted by a polynomial function. The fitting results of peak A and peak B are as follows: $W_{A}=1391.207+0.695Cur-1.812Cur^{2}-4.516Cur^{3}$ with a fitting degree of 0.998, $W_{B}=1591.428+1.398Cur-5.303Cur^{2}+9.909Cur^{3}$ with a fitting degree of 0.994.

When the curvature changes from 0 to 0.915 m^−1^, the maximum wavelength sensitivity of peak A and peak B are −14.028 nm/m^−1^ and 16.392 nm/m^−1^, respectively, and the minimum sensitivities are 0.695 nm/m^−1^ and 0.356 nm/m^−1^, respectively. The wavelength shifts of peak A and peak B are caused by the change in the grating period and effective refractive index of the core and cladding modes during fiber bending, which is related to elastic mechanics and elasto-optical effect. As shown in Ref. [22], the relationship between the resonant wavelength and curvature is related to the waveguide dispersion factor and resonant wavelength, which shows that the curvature sensitivity of the resonant wavelength is not a fixed value. 

### 3.3. Temperature Sensitivity

**Figure 6:**
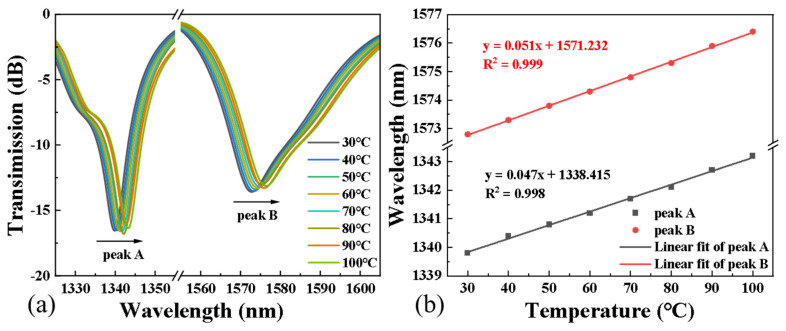
(**a**) The transmission spectrum of the sensor and (**b**) the changes in the wavelength and the fitting results of two resonance peaks with varying temperature.

The spectral response and the corresponding linear fitting of AC-TLPFG at different temperatures are shown (Figure 6a,b). It can be seen that with the increase in temperature, the resonant peaks move to a longer wavelength. The temperature sensitivities of peak A and peak B are 51 pm/°C and 47 pm/°C, respectively. The linear fitting curves are $\lambda_{A}=0.047Tem+1338.415$ with a fitting degree of 0.998 and $\lambda_{B}=0.051Tem+1571.232$ with a fitting degree of 0.999, respectively. From the perspective of the sensing mechanism, when the ambient temperature changes, the fiber length and effective refractive index of the core mode and cladding mode will change due to the thermal expansion effect and thermo-optic effect, and the change in the fiber length will cause the change in the grating period. The changes in these values cause the wavelength shift of the resonant peaks.

### 3.4. Dual-Parameter Sensing

The dual-parameter matrix method is used to measure two environmental parameters simultaneously by detecting the changes in two modulation parameters in the sensor system. For our sensor, the wavelength shifts of peak A and peak B are sensitive to the environmental parameters (curvature and temperature) *Cur* and *Tem* at the same time, so the wavelength drift under different curvatures and temperatures can be expressed as:

(6) $$\Delta \lambda_{Dip\;A}=K_{Cur\mathrm{-A}}\Delta C+K_{Tem\mathrm{-A}}\Delta T,$$

(7) $$\Delta \lambda_{Dip\;B}=K_{Cur\mathrm{-B}}\Delta C+K_{Tem\mathrm{-B}}\Delta T,$$

where *K_Cur_*_-*A*_ and *K_Tem_*_-*A*_ are the bending and temperature sensitivity coefficients of the resonance peak A, respectively, while *K_Cur_*_-*B*_ and *K_Tem_*_-*B*_ are the bending and temperature sensitivity coefficients of the resonant peak B. According to the above formula, the change in the curvature and temperature can be calculated by the change in the wavelength shift of the two peaks, so that the simultaneous measurement of the two parameters can be realized.

The curvature and temperature sensitivity coefficients of the two resonant peaks can be determined by the fitting results in Figure 5 and Figure 6. Therefore, when the curvature and temperature of unknown environmental variables change at the same time, the wavelength shifts Δ*λ_DipA_* and Δ*λ_DipB_* of peak A and peak B can be obtained through appropriate signal demodulation. Curvature and temperature can be determined simultaneously and accurately through the inverse matrix of the sensitivity matrix:

(8) $$\left[\begin{array}{c} \Delta C\\ \Delta T \end{array}\right]={\left[\begin{array}{cc} K_{Cur\mathrm{-A}} & K_{Tem\mathrm{-A}}\\ K_{Cur\mathrm{-B}} & K_{Tem\mathrm{-B}} \end{array}\right]}^{-1}\left[\begin{array}{c} \lambda_{DipA}\\ \lambda_{DipB} \end{array}\right]$$

Table 1 compares our sensor with other reported sensors based on other structures. It shows that our sensor has more advantages than other sensors such as higher curvature sensitivity and two-parameter measurement ability, which shows that our sensor has great potential in engineering applications (Table 1). For example, all optical fiber sensors with such size have practical applications in engineering, such as shape sensing in soft manipulators [23], humanoid robots [24] and endoscopies [25], the human motion and posture monitoring in medical fields [26], and so on.

## 4. Conclusions

In summary, we have reported a novel AC-LPFG sensor with a simultaneous detection ability for curvature and temperature. The sensor was fabricated in our laboratory and consisted of two TLPFGs with different tilt angles. Due to the different angles, two resonant peaks show opposite shifts with increasing curvature and a similar temperature response. By utilizing the different sensitivities of the two peaks to curvature and temperature, a 2 × 2 matrix could be used to demodulate such two parameters simultaneously. The compact size, high sensitivity, and the ability of dual-parameter measurements make our sensor have more potential for engineering applications.

## Figures and Tables

**Table 1:** Comparison of typical bending sensors.

Configuration	Max Curvature Sensitivity (nm/m^−1^)	Range (m^−1^)	Temperature Sensitivity (pm/°C)	Two-Parameter Measurement Ability	Ref.
MCF-based FPI	0.40	0–6.4	176 ± 4	No	[9]
FMF-based MZI	2	0–10	2.34	No	[6]
RCF-based MZI	−3.68	1.3856–3.6661	72	Yes	[11]
Cascaded MZI based on Nano-EYDF	−9.48	0–1.03	94.70	Yes	[5]
Deformed-MMF-based MZI	−23.3	0–1.1733	−52.1	Yes	[14]
HLPFG	1.94	0–9.06	132.8	No	[15]
AR-LPFG	−17.02	0–1.96	92.8	No	[17]
CLPFG	14.08	0.128–1.28	75.6	Yes	[16]
AC-LPFG	16.392	0–0.9125	51	Yes	Our work

## Data Availability

Data are contained within the article.
